# Nursing Leadership Styles and Moral Courage: The Mediating Role of Ethical Dilemma Identification and the Moderating Effects of Ethical Climate and Compassion Fatigue

**DOI:** 10.1155/jonm/9566268

**Published:** 2026-06-28

**Authors:** Mei Xie, Zefeng Shao, Huimin Su, Quanyi Long, Nik Mohd Hazrul Nik Hashim, Kifayat Nahiyan Rafi, Yuanyuan Zou

**Affiliations:** ^1^ Department of Psychology of Developmental and Socialization Processes, Sapienza University of Rome, Rome, Italy, uniroma1.it; ^2^ Graduate School of Business, National University of Malaysia, Bangi, Malaysia, ukm.my; ^3^ Faculty of Economics and Trade, Urumqi Vocational University, Urumqi, Xinjiang, China, uvu.edu.cn; ^4^ Department of Internal Medicine II, The Third Affiliated Hospital of Kunming Medical University, Kunming, China, kmmc.cn; ^5^ School of Nursing, Kunming Medical University, Kunming, China, kmmc.cn; ^6^ Department of Neurosciences, Mental Health and Sensory Organs (NESMOS), Sapienza University of Rome, Rome, Italy, uniroma1.it

**Keywords:** compassion fatigue, ethical climate, ethical dilemma identification, moral courage, nursing leadership

## Abstract

In healthcare organizations, ethical conduct is strongly influenced by nursing leadership. Although prior research has emphasized the role of leadership in shaping professional behavior, limited attention has been given to how different leadership styles affect nurses’ moral courage through ethical decision‐making processes. This study examines the effects of three leadership styles—ethical, empowering, and authoritarian—on nurses’ moral courage, with ethical dilemma identification as a mediator and perceived ethical climate and compassion fatigue as moderators. A cross‐sectional survey was conducted among registered nurses in a Chinese tertiary hospital (*N* = 314). A moderated mediation model was tested using structural equation modeling. The results show that ethical and empowering leadership are positively associated with moral courage, whereas authoritarian leadership is negatively associated. These relationships are mediated by ethical dilemma identification, suggesting that leadership shapes moral courage by influencing nurses’ recognition of ethical issues. Moreover, a supportive ethical climate strengthens the positive effects of ethical and empowering leadership, while compassion fatigue weakens these relationships. These findings highlight the critical role of leadership in promoting ethical practice in nursing. Practically, healthcare organizations should foster ethical and empowering leadership, strengthen ethical climates, and address compassion fatigue to enhance nurses’ moral courage in clinical settings.

## 1. Introduction

Moral courage is a critical component of nursing practice, referring to the ability to act according to ethical principles despite potential personal or professional risks [1, 2]. In complex clinical environments, nurses frequently encounter ethical challenges involving end‐of‐life decisions, resource allocation, and conflicts between patient autonomy and institutional policies [3]. Moral courage is, therefore, essential for maintaining patient safety, ethical decision‐making, and professional integrity [4–6]. However, nurses often face barriers such as hierarchical pressure, fear of punishment, and emotional exhaustion associated with repeated exposure to ethical dilemmas [7].

Leadership plays an important role in shaping nurses’ ethical behavior and responses to ethical challenges [8, 9]. Ethical leadership promotes fairness and integrity, encouraging ethical conduct [10], whereas empowering leadership enhances autonomy and confidence in ethical decision‐making [11]. In contrast, authoritarian leadership (AUL) may suppress ethical discussion and constrain moral agency through rigid control and hierarchical dominance [12]. Although leadership has been widely studied in relation to workplace outcomes, its influence on nurses’ moral courage remains underexplored.

Drawing on ethical decision‐making theory [13], this study proposes that ethical dilemma identification (EDI) serves as a key cognitive mechanism linking leadership styles to moral courage. Leadership may influence nurses’ ability to recognize ethical issues, thereby facilitating or constraining ethical action.

The effects of leadership are also likely to depend on organizational and psychological conditions. A supportive ethical climate may strengthen the positive influence of leadership on ethical awareness, whereas compassion fatigue may weaken this influence by reducing nurses’ cognitive and emotional resources [14–17].

Accordingly, this study develops a moderated mediation model linking ethical, empowering, and AUL to nurses’ moral courage through EDI, with perceived ethical climate (PEC) and compassion fatigue as moderators. The study contributes to the literature in three ways. First, it integrates multiple leadership styles into a unified framework to compare their differential effects on moral courage. Second, it extends ethical decision‐making theory by identifying EDI as a central cognitive mechanism connecting leadership to ethical action. Third, it provides a multilevel explanation of moral behavior in nursing by incorporating both organizational context and individual psychological conditions. In doing so, the study offers theoretical insights and practical guidance for fostering moral courage in contemporary healthcare settings.

## 2. Literature Review and Hypotheses

### 2.1. Nursing Leadership and Moral Courage

Leadership is a critical determinant of ethical behavior in nursing practice, shaping how nurses perceive, interpret, and respond to morally complex situations. Recent studies in nursing contexts have highlighted that leadership styles not only influence clinical performance but also play a key role in fostering ethical awareness, moral courage, and professional accountability among nurses (e.g., [18–21]). Among the many leadership styles, three dimensions—ethical leadership, empowering leadership, and AUL—stand out for their contrasting implications for moral courage.

Ethical leadership emphasizes fairness, integrity, and the consistent role modeling of moral behavior [10]. Leaders who practice ethical leadership not only make principled decisions themselves but also cultivate an environment in which ethical concerns are openly discussed and valued. Prior studies indicate that when leaders act transparently, clarify ethical expectations, and hold themselves accountable, nurses feel more secure in voicing concerns and challenging unethical practices [22]. Such leadership behaviors directly encourage moral courage by signaling that ethical action is institutionally supported and professionally rewarded. One typical obstacle to moral conduct, especially in hierarchical healthcare organizations, is fear of reprisal; however, ethical leadership alleviates this anxiety [23].

In contrast, leaders who practice empowerment aim to increase clinical decision‐making autonomy, involvement, and self‐efficacy [24]. Leaders who empower their nurses do things like include them in decision‐making, distribute responsibility, and trust their expertise [25]. According to studies conducted by Amundsen and Martinsen [26] and Bobbio et al. [27], nurses are more likely to face ethical problems and behave in accordance with their professional principles when they believe they are trusted and competent. Empowering leadership builds moral courage‐supporting psychological resources including accountability, self‐confidence, and resilience by encouraging a feeling of personal ownership and responsibility [28]. When making quick judgments while staying true to their ethical principles, this approach is invaluable for frontline nurses working in unpredictable and fast‐paced healthcare settings.

In contrast, AUL is defined by a strict chain of command, with little room for subordinates to make their own decisions [29]. Leaders with an autocratic style often demand blind obedience, stifle disagreement, and put the sake of efficiency or conformity ahead of ethical discussion. Although this approach may help with compliance and order in certain situations, it often hinders nurses’ moral agency by limiting their chances to assess ethical dilemmas critically [30]. As a result of moral inactivity or silence, nurses working under authoritarian bosses may experience fear of punishment, a lack of agency, or the conviction that their ethical concerns do not matter [31]. The probability of moral bravery among nurses decreases as a result of these situations’ gradual erosion of trust in their ability to face ethical issues.

**Hypothesis 1.**

Ethical leadership positively predicts moral courage.

**Hypothesis 2.**

Empowering leadership positively predicts moral courage.

**Hypothesis 3.**

Authoritarian leadership negatively predicts moral courage.

### 2.2. EDI as a Mediator

Moral courage cannot be enacted unless nurses are first able to recognize that a situation contains an ethical dimension requiring judgment and action. EDI refers to the cognitive process through which healthcare professionals notice, interpret, and evaluate morally problematic situations in clinical practice [32]. According to ethical decision‐making theory [13], the identification of a dilemma is the first step in activating moral reasoning and subsequent action. Without this recognition, even nurses with strong ethical values may remain passive in the face of challenges. Leadership styles can significantly shape this process by either fostering or hindering critical ethical awareness.

Ethical leaders model fairness, transparency, and moral integrity in their interactions [10]. By openly discussing values and highlighting the ethical dimensions of decisions, such leaders help nurses become more attuned to potential moral conflicts in patient care [33]. For example, when leaders frame clinical issues not only in technical but also in ethical terms, nurses learn to recognize subtle conflicts between institutional rules and patient needs. Prior research shows that ethical leadership enhances followers’ moral sensitivity and ethical decision‐making [20]. Thus, ethical leadership is expected to promote nurses’ ability to identify ethical dilemmas, which subsequently enables them to act courageously when moral principles are at stake [18, 19, 21].

Empowering leaders enhance nurses’ autonomy, participation, and confidence in their professional judgment [34, 35]. By involving nurses in decision‐making and encouraging them to critically evaluate different courses of action, empowering leaders cultivate an environment where ethical reflection is a natural part of practice [36]. Nurses who feel trusted and valued are more likely to scrutinize the implications of AI‐generated recommendations, treatment plans, or resource allocations for their ethical consequences. This reflective stance increases the likelihood of recognizing value conflicts in daily care. Previous studies confirm that empowerment fosters critical thinking and ethical awareness in nursing teams [26]. Accordingly, empowering leadership enhances the process of EDI, thereby laying the groundwork for the enactment of moral courage. However, conflicting evidence exists. While empowering leadership has been shown to enhance ethical awareness and autonomy, other studies suggest that it may also increase unethical pro‐organizational behavior under certain conditions [37], indicating that its effects are not uniformly positive.

In contrast, AUL is marked by rigid hierarchies, strict control, and limited opportunities for staff to question or reflect [29]. Under such leadership, nurses may feel pressured to conform to directives without evaluating their ethical implications. AULs often emphasize compliance and obedience [38], which suppresses open discussion and discourages nurses from raising ethical concerns [39]. Over time, this environment diminishes nurses’ sensitivity to moral conflicts, as they internalize the belief that ethical deliberation is neither valued nor rewarded [40]. Studies suggest that authoritarian climates foster moral disengagement and silence, reducing the ability of employees to recognize ethical dilemmas [31]. Thus, AUL may weaken the pathway from ethical awareness to moral courage by obstructing the very first step—identifying that a dilemma exists.

**Hypothesis 4.**

Ethical dilemma identification is positively related to moral courage.

**Hypothesis 5.**

Ethical dilemma identification mediates the relationship between ethical leadership and nurses’ moral courage.

**Hypothesis 6.**

Ethical dilemma identification mediates the relationship between empowering leadership and nurses’ moral courage.

**Hypothesis 7.**

Ethical dilemma identification mediates the relationship between authoritarian leadership and nurses’ moral courage.

### 2.3. Clinical Context Ethical Culture as a Moderator

When trying to deduce the extent to which leadership affects nurses’ ethical conduct, the clinical setting offers crucial boundary conditions. No nurse works in a vacuum; the ethical culture of their workplace influences both their thoughts and their deeds. Significant modifiers include the perceived ethical atmosphere and compassion weariness, two contextual variables. A supportive ethical atmosphere may enhance leadership’s impact on identifying ethical dilemmas, but when nurses experience high levels of compassion fatigue, it can drain their psychological resources and undermine these impacts.

PEC refers to the shared perception among staff that ethical values, fairness, and integrity are emphasized, supported, and rewarded within the workplace [15, 17]. In such climates, nurses feel safe to voice concerns, question practices, and engage in moral deliberation without fear of reprisal. Previous research shows that positive ethical climates foster ethical awareness, job satisfaction, and commitment to professional standards [41]. When paired with ethical or empowering leadership, a supportive ethical climate further legitimizes ethical reflection and signals that identifying moral conflicts is not only appropriate but expected [42]. For instance, ethical leaders who promote fairness will have an even stronger effect on nurses’ recognition of ethical dilemmas when the organization also reinforces values of justice and patient‐centered care. Thus, PEC strengthens the pathway between supportive leadership and EDI, amplifying the likelihood that nurses will engage in courageous moral action [43].

In contrast, compassion fatigue represents a significant psychological barrier that undermines the positive influence of leadership. Compassion fatigue is a form of emotional and moral exhaustion that results from prolonged exposure to patient suffering and repeated ethical stressors [14, 16]. Nurses experiencing high levels of compassion fatigue often report diminished empathy, decreased attentiveness, and emotional detachment, all of which impair their ability to recognize ethical issues [5]. Even when guided by ethical or empowering leaders, nurses who are emotionally depleted may lack the cognitive and affective resources necessary to critically evaluate morally complex situations. According to research conducted by Hinderer et al. [44], those who experience compassion fatigue tend to be less sensitive to ethical issues, less committed to their careers, and more morally disengaged. Consequently, leadership may not inspire moral bravery as much as it otherwise would since compassion fatigue mitigates the positive effect of leadership on recognizing ethical dilemmas.

**Hypothesis 8.**

Perceived ethical climate strengthens the relationship between ethical leadership and ethical dilemma identification.

**Hypothesis 9.**

Perceived ethical climate strengthens the relationship between empowering leadership and ethical dilemma identification.

**Hypothesis 10.**

Perceived ethical climate strengthens the relationship between authoritarian leadership and ethical dilemma identification.

**Hypothesis 11.**

Compassion fatigue weakens the relationship between ethical leadership and ethical dilemma identification.

**Hypothesis 12.**

Compassion fatigue weakens the relationship between empowering leadership and ethical dilemma identification.

**Hypothesis 13.**

Compassion fatigue strengthens the relationship between AUL and ethical dilemma identification.

**FIGURE 1:**
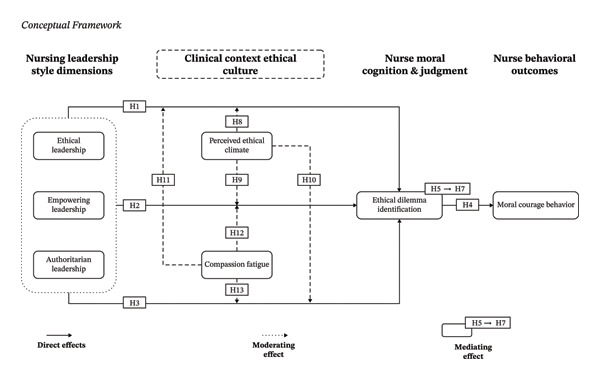
Conceptual framework. *Source(s):* Author’s own work.

The conceptual framework is shown in Figure 1.

Taken together, existing studies provide valuable insights into the role of leadership in shaping ethical behavior; however, several limitations remain. First, findings across studies are not always consistent, particularly regarding the effects of empowering leadership, which may produce both positive and unintended consequences. Second, much of the literature has focused on general organizational outcomes, with limited attention to ethical cognition as a mediating mechanism. Third, contextual and psychological boundary conditions have often been overlooked. These gaps highlight the need for a more integrative approach that simultaneously considers leadership styles, cognitive processes, and contextual factors, as proposed in the present study.

## 3. Methodology

### 3.1. Design and Sample

This study adopted a cross‐sectional survey design to investigate the relationships among nursing leadership styles, EDI, PEC, compassion fatigue, and moral courage behavior (MCB). The study was reported in accordance with the STROBE statement for cross‐sectional observational research. Data were collected in August 2025 from registered nurses working at an affiliated hospital of a medical university, a large tertiary‐level teaching hospital in Yunnan Province, China. A convenience sampling approach was employed to ensure participation from diverse clinical departments, including internal medicine, surgery, emergency care, and intensive care units. Inclusion criteria were as follows: (1) being a registered nurse, (2) having at least 6 months of clinical work experience, and (3) currently working in a clinical department of the participating hospital. Nurses on long‐term leave or administrative staff without direct clinical responsibilities were excluded from the study. A total of 350 questionnaires were distributed through a widely used online survey platform in China, and 314 valid responses were obtained after excluding incomplete questionnaires, yielding an effective response rate of 89.7%. The final sample size exceeded the minimum recommendations for partial least squares structural equation modeling (PLS‐SEM). Following the commonly applied “10‐times rule,” the required sample size should be at least ten times the maximum number of structural paths directed at any endogenous construct in the model [45]. Given the complexity of the proposed moderated mediation model, the sample size of 314 was considered adequate to ensure sufficient statistical power and stable parameter estimation. All retained questionnaires were fully completed, and therefore, no missing data were present in the final dataset used for analysis. Ethical approval for the study was obtained, and permission was granted by the affiliated hospital. Participation was voluntary, with assurances of anonymity and confidentiality. The study protocol and analysis plan were not preregistered prior to data collection. However, all analyses were conducted in accordance with the study objectives and established methodological procedures.

### 3.2. Demographic Characteristics

Table 1 presents the demographic characteristics of the participating nurses. The sample included a majority of female nurses, most of whom were between 18 and 39 years of age, with varied educational backgrounds, professional titles, and years of work experience. Representation was achieved across major clinical departments, ensuring the sample reflected diverse nursing contexts.

**TABLE 1:** Respondent’s demographic characteristics (*N* = 314).

Variable	Category	Frequency	Percentage (%)
Age	18–29	90	28.66
	30–39	159	50.64
	40 and above	65	20.70

Gender	Male	16	5.10
	Female	298	94.90

Marital status	Single	61	19.43
	Married	239	76.11
	Other	14	4.46

Education	Secondary nursing diploma	65	20.70
	Junior college diploma	105	33.44
	Bachelor’s degree	135	42.99
	Master’s degree	9	2.87

Professional title	Intern nurse	36	11.5
	Advanced training nurse	34	10.8
	Resident nurse (standardized training nurse)	71	22.6
	Nurse	112	35.7
	Senior nurse	35	11.1
	Charge nurse	16	5.1
	Associate chief nurse	8	2.5
	Chief nurse	2	0.6

Job position	General clinical nursing staff	198	63.1
	Nursing team leader/charge nurse	67	21.3
	Head nurse	24	7.6
	Nursing department staff	25	8.0

Years of experience	≤ 5 years	58	18.47
	6–10 years	156	49.68
	11–20 years	73	23.25

### 3.3. Measures

All constructs in this study were assessed using well‐established and validated scales, with minor adaptations to ensure contextual relevance for Chinese nurses (see Table A1 in Appendix). Unless otherwise indicated, items were rated on a five‐point Likert scale ranging from 1 (*strongly disagree*) to 5 (*strongly agree*). For certain constructs (e.g., AUL and compassion fatigue), original scale formats with six‐point or alternative response anchors were retained to preserve the validated structure and psychometric properties of the instruments. Maintaining original response formats is consistent with prior research and ensures comparability with existing studies. Translation and back‐translation procedures were applied to ensure semantic and conceptual equivalence of all measures, followed by pilot testing with 30 nurses to ensure clarity and contextual relevance.

#### 3.3.1. Ethical Leadership

Ethical leadership was measured using the improved scale developed by Yukl et al. [46]. This instrument assesses leaders’ fairness, honesty, concern for subordinates, and consistency in modeling ethical standards. A sample item is *“My supervisor sets an example of ethical behavior in his/her decisions and actions.”* Higher scores indicate stronger perceptions of ethical leadership.

#### 3.3.2. Empowering Leadership

Empowering leadership was assessed with the Empowering Leadership Questionnaire (ELQ) developed by Arnold et al. [24]. The ELQ evaluates leader behaviors that foster autonomy, encourage participation in decision‐making, and enhance confidence in subordinates’ abilities. Example items include *“My supervisor encourages me to express my ideas and suggestions”* and *“My supervisor shows confidence in my ability to perform at a high level.”* The ELQ has demonstrated strong psychometric properties in previous organizational studies [47].

#### 3.3.3. AUL

AUL was measured using the nine‐item subscale developed by Cheng et al. [29]. This scale captures two dimensions: Zhuanquan (use of authority to demand compliance) and Shangyan (strict discipline and emphasis on high performance). A sample item is *“Our supervisor determines all decisions in the organization whether they are important or not.”* Responses were given on a six‐point Likert scale ranging from 1 (*very seldom*) to 6 (*very frequent*). Prior research has reported high reliability for this scale [48].

#### 3.3.4. PEC

Nurses’ perceptions of the ethical climate in their workplace were measured using items adapted from the Ethical Climate Questionnaire [49], which has been widely applied and validated in diverse organizational and cultural settings, including studies conducted in China. To further ensure cultural appropriateness in the nursing context, items were refined to reflect ethical norms and organizational practices specific to Chinese hospitals. The scale demonstrated satisfactory reliability and validity in the present study (see Table 2). This measure captures perceptions of fairness, integrity, and the degree to which ethical behavior is encouraged and supported by the organization. Respondents indicated their agreement with statements such as *“In this organization, ethical behavior is rewarded.”*

**TABLE 2:** Convergent validity.

Variable	Item	Standardized factor loadings	Cronbach’s alpha	Composite reliability (rho_a)	Composite reliability (rho_c)	AVE	VIF
Ethical leadership	ETL‐1	0.766	0.929	0.930	0.939	0.561	2.139
	ETL‐2	0.736					1.959
	ETL‐3	0.740					1.997
	ETL‐4	0.771					2.215
	ETL‐5	0.764					2.099
	ETL‐6	0.753					1.999
	ETL‐7	0.736					1.913
	ETL‐8	0.715					1.821
	ETL‐9	0.755					2.038
	ETL‐10	0.766					2.053
	ETL‐11	0.726					1.937
	ETL‐12	0.758					1.992

Empowering leadership	EML‐1	0.818	0.874	0.877	0.903	0.571	2.230
	EML‐2	0.761					1.771
	EML‐3	0.757					1.740
	EML‐4	0.723					1.660
	EML‐5	0.721					1.713
	EML‐6	0.724					1.650
	EML‐7	0.779					1.983

Authoritarian leadership	AL‐1	0.823	0.923	0.927	0.936	0.618	2.447
	AL‐2	0.840					2.563
	AL‐3	0.759					2.004
	AL‐4	0.773					2.090
	AL‐5	0.768					2.043
	AL‐6	0.761					1.919
	AL‐7	0.758					2.003
	AL‐8	0.789					2.119
	AL‐9	0.801					2.312

Moral courage behavior	MCB‐1	0.765	0.915	0.916	0.929	0.566	2.030
	MCB‐2	0.734					1.868
	MCB‐3	0.746					1.918
	MCB‐4	0.720					1.789
	MCB‐5	0.767					1.992
	MCB‐6	0.720					1.820
	MCB‐7	0.757					1.978
	MCB‐8	0.753					1.909
	MCB‐9	0.776					2.028
	MCB‐10	0.782					2.163

Ethical dilemma identification	EDI‐1	0.785	0.936	0.937	0.945	0.588	2.333
	EDI‐2	0.788					2.323
	EDI‐3	0.783					2.295
	EDI‐4	0.757					2.095
	EDI‐5	0.821					2.640
	EDI‐6	0.759					2.063
	EDI‐7	0.735					1.893
	EDI‐8	0.718					1.844
	EDI‐9	0.748					1.977
	EDI‐10	0.760					2.037
	EDI‐11	0.776					2.258
	EDI‐12	0.764					2.182

Perceived ethical climate	PEC‐1	0.823	0.933	0.937	0.943	0.600	2.659
	PEC‐2	0.795					2.119
	PEC‐3	0.795					2.168
	PEC‐4	0.776					2.155
	PEC‐5	0.765					2.106
	PEC‐6	0.776					1.951
	PEC‐7	0.755					1.985
	PEC‐8	0.758					2.231
	PEC‐9	0.746					2.602
	PEC‐10	0.752					2.369
	PEC‐11	0.776					2.310

Compassion fatigue	CF‐1	0.851	0.949	0.961	0.956	0.684	3.140
	CF‐2	0.865					3.126
	CF‐3	0.832					2.781
	CF‐4	0.810					2.721
	CF‐5	0.827					2.778
	CF‐6	0.796					2.164
	CF‐7	0.817					2.437
	CF‐8	0.789					2.333
	CF‐9	0.823					2.732
	CF‐10	0.856					3.300

#### 3.3.5. Compassion Fatigue

Compassion fatigue was assessed using the Professional Quality of Life Scale (ProQOL), originally developed by Stamm [50] and later validated in Turkish by Yesil et al. [51]. The ProQOL consists of three subscales: compassion satisfaction, burnout, and compassion fatigue. In this study, only the compassion fatigue subscale was used, comprising 10 items (e.g., “*I feel overwhelmed by the suffering of patients I care for*”). Items were rated on a five‐point scale ranging from 0 (*never*) to 5 (*very frequently*). Scores of 22 or below indicate low compassion fatigue, 23–41 moderate, and 42 or above high.

#### 3.3.6. EDI

EDI was measured using items adapted from the Moral Distress‐Appraisal Scale developed by Baele and Fontaine [52]. Although this scale was originally developed in a Western context, prior studies have demonstrated its applicability in healthcare settings across different cultural contexts. In the present study, to ensure cultural appropriateness for Chinese nurses, items were carefully adapted to reflect common ethical situations in local clinical practice. The scale demonstrated satisfactory reliability and construct validity (see Table 2). Example items include *“I can recognize when clinical decisions raise ethical concerns.”* Higher scores reflect stronger EDI.

#### 3.3.7. MCB

Moral courage was assessed with the Nurses’ Moral Courage Scale (NMCS) developed and validated by Numminen et al. [53]. The NMCS comprises 21 items across four dimensions: compassion and true presence (5 items), moral responsibility (4 items), moral integrity (7 items), and commitment to providing good care (5 items). Each item is rated on a five‐point Likert scale ranging from 1 (does not describe me at all) to 5 (describes me very well). A sample item is *“I am willing to defend patient rights even when it is difficult.”* Higher scores indicate greater self‐assessed moral courage.

### 3.4. Data Analysis

PLS‐SEM using SmartPLS 4.0 software was employed to analyze the data. This approach was selected for several reasons specific to the present study. First, the proposed model is relatively complex, incorporating multiple leadership styles alongside both mediating (EDI) and moderating variables (PEC and compassion fatigue), which PLS‐SEM handles efficiently. Second, the study adopts a predictive and exploratory orientation, aiming to explain variance in moral courage rather than to test a well‐established theoretical model, making PLS‐SEM more appropriate than covariance‐based SEM. Third, given the moderate sample size and the potential for non‐normality in self‐reported survey data, PLS‐SEM provides a robust estimation approach. Together, these considerations support the suitability of PLS‐SEM for the analytical objectives of this study. Evaluation of the measurement model, including indicator reliability, internal consistency reliability, convergent validity, and discriminant validity, was the first step in the two‐stage process. The second step involved evaluating the structural model, which included path coefficients, coefficient of determination (*R* ^2^), effect size (*f* ^2^), and predictive relevance (*Q* ^2^). In order to evaluate the importance of indirect and conditional effects, bootstrapping with 5000 resamples was used to examine moderate mediation effects. Although demographic variables such as age, gender, education level, and years of experience were collected, they were not included as control variables in the structural model. This decision was based on the study’s primary focus on theory‐driven relationships among leadership styles, EDI, and moral courage, as well as prior research suggesting that demographic variables show inconsistent effects on these constructs. Nevertheless, the omission of control variables may limit the ability to fully rule out alternative explanations.

## 4. Analysis and Results

### 4.1. Measurement Model

PLS‐SEM in SmartPLS 4.0 was used to analyze the measurement model in order to determine the validity and reliability of the reflecting constructs. PLS‐SEM was selected due to its capacity to handle exploratory research in nursing and organizational settings, non‐normal data, and complicated moderated mediation models [45]. Standardized outer loadings were used to assess indicator reliability; all of them were over the 0.70 threshold, indicating that each item sufficiently converged on its construct, as shown in Table 2 [45]. Cronbach’s alpha (*α*), rho (*a*), rho (*c*), and composite reliability (CR) were used to confirm internal consistency dependability; all of these metrics were above the 0.70 threshold [45, 54] (see Table 2).

Convergent validity was established by the average variance extracted (AVE), with all values above the 0.50 cutoff, demonstrating that the constructs accounted for more than half of the variance in their indicators [55]. AVE scores varied from 0.561 (ethical leadership) to 0.684 (compassion fatigue), supporting adequate convergent validity. Additionally, variance inflation factor (VIF) values were calculated to assess collinearity among indicators, all below the 3.3 threshold (ranging from 1.650 to 3.300), confirming no multicollinearity issues at the item level [56] (see Table 2).

Discriminant validity was confirmed using the Heterotrait–Monotrait (HTMT) ratio and Fornell–Larcker criterion, as presented in Table 3. For the Fornell–Larcker criterion, the square root of AVE (diagonal values: e.g., 0.786 for AUL and 0.767 for EDI) exceeded all interconstruct correlations (off‐diagonals: e.g., −0.487 between AUL and EDI, and 0.553 between EDI and MCB), indicating that constructs were more strongly related to their own indicators than to others [55]. For the HTMT criterion, all values were below the conservative 0.85 threshold (e.g., 0.518 between AUL and EDI, 0.594 between EDI and MCB, and 0.367 between AUL and PEC), further confirming that the constructs were empirically distinct [57]. These results validate the measurement model’s quality, ensuring reliable progression to structural path estimation.

**TABLE 3:** Discriminant validity.

	1	2	3	4	5	6	7
Fornell and Larcker criterion							
AUL	0.786						
EDI	−0.487	0.767					
EML	−0.450	0.519	0.756				
ETL	−0.555	0.465	0.477	0.749			
MCB	−0.363	0.553	0.377	0.340	0.752		
MF	0.165	−0.188	−0.092	−0.106	−0.155	0.827	
PEC	−0.344	0.293	0.235	0.299	0.232	−0.348	0.774

Heterotrait–Monotrait (HTMT) criterion							
AUL							
EDI	0.518						
EML	0.497	0.572					
ETL	0.597	0.495	0.530				
MCB	0.392	0.594	0.419	0.366			
MF	0.173	0.192	0.108	0.110	0.158		
PEC	0.367	0.308	0.259	0.317	0.245	0.368	

Abbreviations: AUL, authoritarian leadership; CF, compassion fatigue; EDI, ethical dilemma identification; EML, empowering leadership; ETL, ethical leadership; MCB, moral courage behavior; PEC, perceived ethical climate.

### 4.2. Common Method Bias (CMB)

Given the self‐reported data from a single survey, CMB was addressed through both procedural and statistical procedures [58]. Procedurally, participants were assured of anonymity and voluntary participation to reduce social desirability effects. The survey sequence separated independent variables (e.g., leadership styles) and dependent variables (e.g., MCB) with demographic filler items, and instructions emphasized honest responses without right or wrong answers. Scales were adapted for cultural relevance in the Chinese context via back‐translation and pretested on a pilot sample of 30 nurses for clarity.

Statistically, Harman’s single‐factor test via exploratory factor analysis extracted a principal component explaining 22.231% of the variance, below the 50% threshold, suggesting that no single factor accounted for the majority of covariance [58, 59]. However, given the limitations of this test, additional assessments were conducted. Specifically, a full collinearity test based on VIFs was performed, as recommended for PLS‐SEM analyses. All VIF values were below the conservative threshold of 3.3, indicating that CMB is unlikely to pose a serious threat to the results [56].

### 4.3. Structural Model

Model fit indices generally indicated an acceptable level of fit. The standardized root mean square residual (SRMR = 0.045) was below the recommended threshold of 0.08, suggesting a good fit. The discrepancy measures (d_ULS = 5.206; d_G = 1.865) were within acceptable ranges. However, the normed fit index (NFI = 0.814) was slightly below the commonly suggested benchmark of 0.90, indicating a marginal fit for this index [60]. Taken together, these results suggest that the model demonstrates an overall acceptable, though not perfect, fit. Consistent with recent recommendations, model fit was evaluated using multiple indices rather than relying on a single criterion, and the findings should be interpreted with appropriate caution.

Explanatory power was assessed via *R* ^2^ values. The model explained 30.4% of the variance in MCB (*R* ^2^ = 0.304) and 59.5% in EDI (*R* ^2^ = 0.595). According to established benchmarks [61], these values indicate a moderate level of explanatory power for moral courage and a substantial level for EDI. This suggests that leadership styles and contextual factors play a meaningful role in shaping nurses’ ethical cognition and behavior, particularly in influencing their ability to recognize ethical dilemmas. Predictive relevance was supported by *Q* ^2^ values > 0 (*Q* ^2^ = 0.205 for MCB; 0.551 for EDI) from the blindfolding procedure [62]. Effect size (*f* ^2^) analysis further clarified the relative importance of predictors. Ethical leadership showed a small effect on EDI (*f* ^2^ = 0.035), while empowering leadership demonstrated a medium effect (*f* ^2^ = 0.171), indicating a comparatively stronger influence. AUL exerted a small negative effect (*f* ^2^ = 0.067). Notably, EDI had a large effect on MCB (*f* ^2^ = 0.441), suggesting that it serves as a central mechanism linking leadership to behavioral outcomes (see Table 4). These findings highlight that, among the examined predictors, empowering leadership and EDI play particularly important roles in shaping nurses’ moral courage.

**TABLE 4:** Structural model fit indices.

	1	2	3	4	5	Model fit indices
*R* ^2^	—	—	—	0.595	0.304	
*Q* ^2^	—	—	—	0.551	0.205	
*f* ^2^	—	—	—			
1. Ethical leadership	—	—	—	0.035	—	
2. Empowering leadership	—	—	—	0.171	—	
3. Authoritarian leadership	—	—	—	0.067	—	
4‐Ethical dilemma identification	—	—	—	—	0.441	
5‐Moral courage behavior	—	—	—	—	—	
SRMR	—	—	—			0.045
d_ULS	—	—	—			5.206
d_G	—	—	—			1.865
Chi‐square						2952.761
NFI	—	—	—			0.814

Path analyses supported most direct effects. Ethical leadership (*β* = 0.153, *p* < 0.01) and empowering leadership (*β* = 0.310, *p* < 0.001) positively predicted EDI, while AUL showed a negative effect (*β* = −0.206, *p* < 0.001), confirming H1, H2, and H3. EDI positively influenced MCB (*β* = 0.553, *p* < 0.001; see Table 5).

**TABLE 5:** Hypothesis testing.

Hypotheses	Relationship	*β*	Confidence intervals	STDEV	*T*‐value	*p* value	Relationship
Direct effect							
H1	ETL ⟶ EDI	0.153	(0.063, 0.260)	0.060	2.557	*p* < 0.01	Accepted
H2	EML ⟶ EDI	0.310	(0.223, 0.397)	0.053	5.876	*p* < 0.001	Accepted
H3	AUL ⟶ EDI	−0.206	(−0.299, −0.110)	0.057	3.579	*p* < 0.001	Accepted
H4	EDI ⟶ MCB	0.553	(0.485, 0.628)	0.044	12.695	*p* < 0.001	Accepted
Mediating effect							
H5	ETL ⟶ EDI ⟶ MCB	0.085	(0.005, 0.038)	0.035	2.436	*p* < 0.01	Accepted
H6	EML ⟶ EDI ⟶ MCB	0.171	(0.008, 0.044)	0.034	5.016	*p* < 0.001	Accepted
H7	AUL ⟶ EDI ⟶ MCB	−0.114	(0.007, 0.040)	0.033	3.429	*p* < 0.001	Accepted
Moderating effect							
H8	PEC ∗ ETL ⟶ EDI	0.166	(0.062, 0.278)	0.066	2.531	*p* < 0.01	Accepted
H9	PEC ∗ EML ⟶ EDI	0.287	(0.186, 0.381)	0.06	4.793	*p* < 0.001	Accepted
H10	PEC ∗ AUL ⟶ EDI	0.214	(0.094, 0.338)	0.074	2.891	*p* < 0.01	Accepted
H11	CF ∗ ETL ⟶ EDI	−0.119	(−0.224, −0.003)	0.067	1.783	*p* < 0.05	Accepted
H12	CF ∗ EML ⟶ EDI	−0.119	(−0.194, −0.033)	0.05	2.385	*p* < 0.01	Accepted
H13	CF ∗ AUL ⟶ EDI	0.015	(−0.107, 0.146)	0.078	0.196	*p* > 0.05	Rejected

*Note:* ^∗^ *p* ≤ 0.05, ^∗∗^ *p* ≤ 0.01, ^∗∗∗^ *p* ≤ 0.001.

Abbreviations: AUL, authoritarian leadership; CF, compassion fatigue; EDI, ethical dilemma identification; ETL, ethical leadership; EML, empowering leadership; MCB, moral courage behavior; PEC, perceived ethical climate; STDEV, standard deviation.

Mediation tests via bootstrapping indicated significant indirect effects. EDI mediated the ethical leadership–MCB path (*β* = 0.085, *p* < 0.01; full mediation) and empowering leadership path (*β* = 0.171, *p* < 0.001), but negative mediated the relationship between AUL and MCB (*β* = −0.114, *p* < 0.001), supporting H5, H6, and H7 ([63]; see Table 5).

**FIGURE 2:**
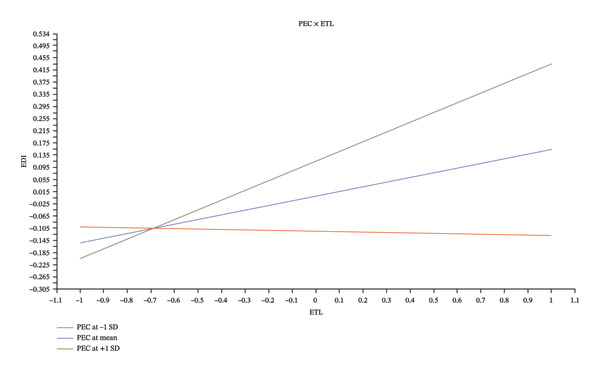
Simple slopes of the moderating effect of perceived ethical climate on the relationship between ethical leadership and ethical dilemma identification.

**FIGURE 3:**
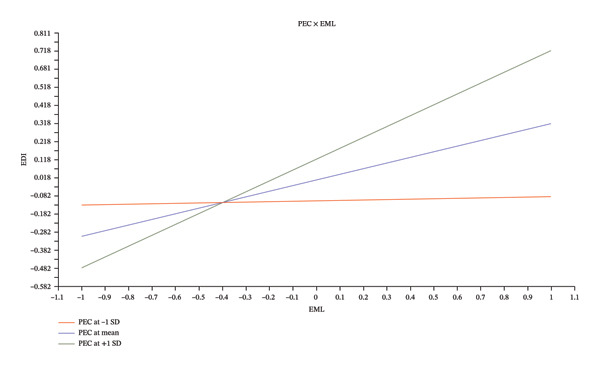
Simple slopes of the moderating effect of perceived ethical climate on the relationship between empowering leadership and ethical dilemma identification.

**FIGURE 4:**
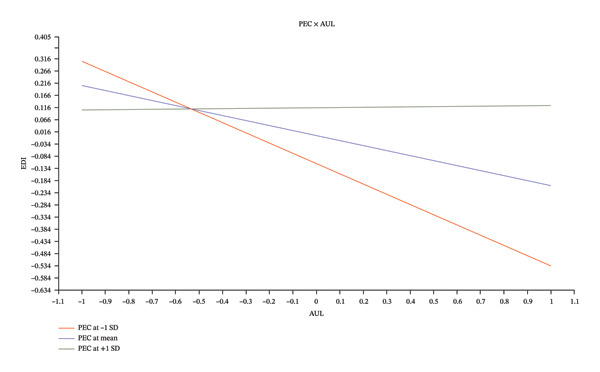
Simple slopes of the moderating effect of perceived ethical climate on the relationship between authoritarian leadership and ethical dilemma identification.

**FIGURE 5:**
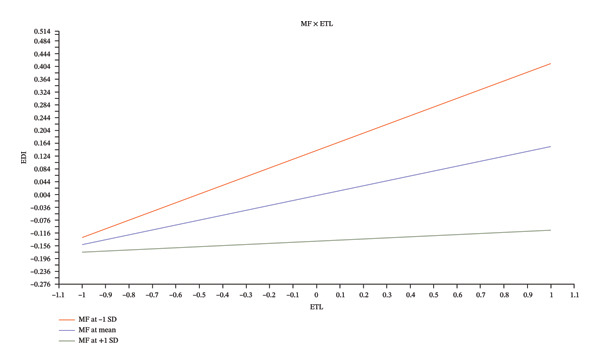
Simple slopes of the moderating effect of compassion fatigue on the relationship between ethical leadership and ethical dilemma identification.

**FIGURE 6:**
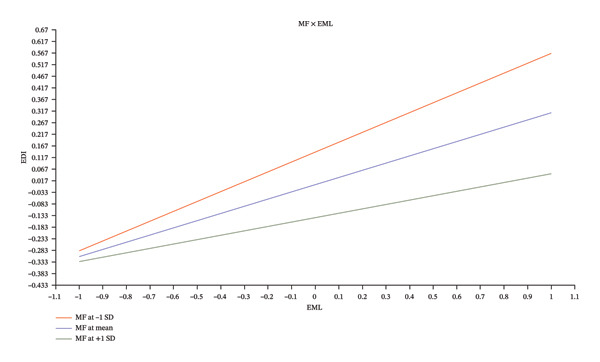
Simple slopes of the moderating effect of compassion fatigue on the relationship between empowering leadership and ethical dilemma identification.

Moderation analyses revealed that PEC strengthened the ethical leadership–EDI link (*β* = 0.166, *p* < 0.01), empowering leadership link (*β* = 0.287, *p* < 0.001) and authoritarian path (*β* = 0.214, *p* < 0.01), supporting H8, H9, and H10. Conversely, compassion fatigue weakened the ethical leadership (*β* = −0.119, *p* < 0.05) and empowering leadership paths (*β* = −0.119, *p* < 0.01) to EDI, supporting H11 and H12. However, compassion fatigue did not moderate the relationship between AUL and EDI (*β* = 0.015, *p* = 0.442), rejecting H13. Simple slopes analysis (Figures 2–6) showed stronger positive effects at high PEC (+1 SD) versus low (−1 SD) and attenuated effects at high compassion fatigue (+1 SD) versus low (−1 SD) [64].

### 4.4. Importance–Performance Map Analysis (IPMA)

**FIGURE 7:**
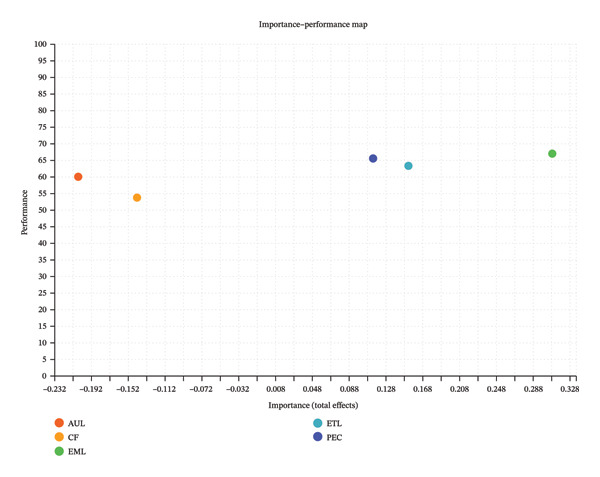
Importance–performance map analysis.

To derive managerial implications, an IPMA was conducted in SmartPLS 4.0, extending the PLS‐SEM results by comparing the importance (total effects) and performance (mean scores, rescaled to 0–100) of predictors on MCB [45, 65]. As shown in Table 6, empowering leadership emerged as the most important driver (importance = 0.310) and performance index (66.895), suggesting prioritization for enhancement through targeted empowering leadership. Ethical leadership ranked second in importance (0.153) but second in performance (66.290), indicating untapped potential for ethical leadership development initiatives. The IPMA plot (Figure 7) positions EDI and ethical leadership in the “high importance, low performance” quadrant, advising hospital administrators to focus on cognitive ethics workshops. Variables such as PEC fall in the “low importance, high performance” area, ideal for resource‐efficient sustainment. These insights guide prioritization in nursing organizations to optimize leadership’s impact on moral courage [45].

**TABLE 6:** Importance–performance map analysis (IPMA) on impulse buying behavior in ethical dilemma identification.

Variables	Importance index	Performance index
Ethical leadership (ETL)	0.153	63.290
Empowering leadership (EML)	0.310	66.895
Authoritarian leadership (AUL)	−0.206	59.967

## 5. Discussion

### 5.1. General Findings

The findings highlight the central role of leadership in shaping nurses’ moral courage and ethical behavior [8, 9]. Ethical, empowering, and AUL demonstrated distinct effects on moral courage, both directly and indirectly through EDI, while contextual factors further influenced these relationships.

Ethical leadership was positively associated with nurses’ moral courage. Nurses who perceived higher levels of ethical leadership reported stronger EDI, which in turn enhanced moral courage. This finding supports previous research suggesting that ethical leaders foster environments characterized by fairness, integrity, and ethical support [10, 22]. Moreover, the positive effect of ethical leadership became stronger under supportive ethical climates but weaker under high levels of compassion fatigue, indicating that emotional exhaustion may reduce nurses’ ability to translate ethical awareness into courageous action [14, 16, 43].

Empowering leadership also positively predicted moral courage, both directly and indirectly through EDI. Among the three leadership styles, empowering leadership showed the strongest effect, suggesting that autonomy, participation, and trust in professional judgment are particularly important for encouraging ethical action in nursing practice [24, 26]. Similar to ethical leadership, the positive effects of empowering leadership were strengthened by supportive ethical climates and weakened by compassion fatigue. These findings suggest that empowerment is most effective when nurses have sufficient organizational support and emotional resources [34, 36, 47].

In contrast, AUL negatively influenced moral courage and reduced nurses’ ability to identify ethical dilemmas. This finding aligns with previous studies showing that rigid hierarchies and controlling leadership suppress ethical sensitivity and discourage moral action [29, 39, 40]. Unlike the other leadership styles, compassion fatigue did not significantly moderate this relationship, suggesting that the negative influence of AUL may operate consistently regardless of nurses’ emotional state. Although supportive ethical climates partially buffered this negative effect, AUL remained detrimental overall [17, 48].

Overall, the findings support ethical decision‐making theory [13] by demonstrating that EDI serves as an important cognitive mechanism linking leadership to moral courage. Ethical and empowering leadership enhanced nurses’ recognition of ethical issues, whereas AUL weakened this process. At the same time, ethical climate and compassion fatigue acted as important contextual conditions shaping these relationships [20, 32].

Several relationships in the model showed relatively small effect sizes, particularly the effects of ethical and AUL on EDI. This may reflect the complex nature of ethical cognition in nursing, which is likely influenced not only by leadership but also by individual and organizational factors such as moral identity, professional experience, and institutional constraints [5, 18].

The findings should also be interpreted with caution. Because the study relied on cross‐sectional self‐report data, alternative explanations such as reverse causality and perceptual bias cannot be excluded [58]. For example, nurses with stronger moral courage may be more likely to perceive leadership positively. Future research using longitudinal, experimental, or multi‐source designs is therefore needed to further validate the proposed relationships.

### 5.2. Implications

#### 5.2.1. Theoretical Implications

Ethics in nursing, leadership, and organizational behavior are all areas that benefit greatly from the study’s theoretical contributions. As a first contribution to the research on moral bravery, it shows how different leadership styles affect ethical conduct in two ways: directly, via the identification of ethical dilemmas, and indirectly, through other means. Unlike previous research that tended to see moral bravery as an immutable quality of character or an essential part of one’s professional identity [66], this study places moral courage in the context of ever‐changing organizational dynamics. This shift highlights that moral courage is not only an individual characteristic but also a contextually nurtured outcome that can be shaped by leadership and organizational culture.

Second, the study integrates organizational behavior theories into the nursing ethics literature. By examining ethical, empowering, and AUL styles, it highlights the differentiated effects of leadership on ethical awareness and courage. Ethical leadership provides role modeling and transparency, empowering leadership fosters autonomy and confidence, and AUL suppresses ethical sensitivity. In doing so, the study contributes to a nuanced understanding of leadership as a multidimensional construct with both enabling and constraining effects on ethical behavior.

Third, by demonstrating the mediating role of EDI, the study connects nursing ethics with decision‐making theories [13]. It provides empirical support for the argument that ethical action requires the prior recognition of a dilemma, thereby reinforcing the theoretical model that ethical behavior unfolds in sequential stages. This contributes to bridging the gap between abstract ethical theories and practical decision‐making models in nursing.

Finally, the moderated mediation design extends sociotechnical systems theory by illustrating how organizational (ethical climate) and psychological (compassion fatigue) contexts condition the leadership–ethics relationship. This multilevel integration underscores that ethical behavior cannot be understood solely at the individual level; instead, it emerges from the intersection of leadership practices, organizational structures, and personal well‐being.

#### 5.2.2. Practical Implications

From a practical perspective, the findings yield several actionable insights for nursing management and healthcare organizations. To begin, there is mounting evidence that nursing practice would benefit from more ethical and empowering leadership styles. Fairness, openness, ethical modeling, transfer of power, and staff engagement in decision‐making should be emphasized in leadership training and professional development programs. Organizational moral courage is strengthened when leaders exhibit these traits because they create settings where nurses feel empowered to recognize and address ethical challenges. Notably, empowering leadership emerged as the strongest predictor of moral courage among the three styles, indicating that strategies which prioritize autonomy and trust in clinical judgment should receive particular attention in leadership development initiatives.

Second, the research emphasizes the significance of healthcare institutions fostering ethical cultures that are helpful. Policies and procedures should be put in place by hospital administrators to encourage open discussion of ethical issues, recognize and promote ethical actions, and incorporate ethical considerations into everyday decision‐making. Some practical ways to improve ethical cultures include forming ethics committees, providing frequent ethics training, and empowering nurses to raise moral issues freely. These endeavors not only strengthen leadership’s beneficial benefits but they also embed ethical reflection into the fabric of the business.

Thirdly, the results highlight the critical need of addressing compassion fatigue immediately, as it has the potential to deplete nurses’ emotional reserves and reduce the impact of leadership that is supportive. Workload management, resilience training, psychological therapy, and peer support groups are some of the tactics that healthcare organizations could use to avoid or lessen the impact of compassion fatigue. Organizations may keep their nurses’ emotional and cognitive capacities to identify ethical challenges and act bravely by emphasizing their well‐being.

Finally, nursing education and ongoing professional development should explicitly integrate training on ethical decision‐making and moral courage. Simulation exercises, case‐based learning, and interprofessional ethics workshops can help nurses practice identifying ethical dilemmas and responding with courage in controlled environments. Over time, such initiatives can translate into improved ethical sensitivity and resilience in real‐world clinical contexts.

### 5.3. Limitations and Future Research

Despite its contributions, this study has several limitations. First, the cross‐sectional design prevents causal inferences. Although the findings support the proposed relationships, the directionality of effects cannot be definitively established. For example, nurses with higher moral awareness may be more likely to perceive leadership behaviors positively. Future longitudinal or experimental studies are, therefore, needed to clarify causal pathways and examine how these relationships evolve over time.

Second, the study relied on convenience sampling from a single tertiary hospital in Yunnan Province, China, which limits the generalizability of the findings. Selection bias may also exist, as participation was voluntary and may have attracted nurses who were more ethically engaged or willing to express their views. In addition, cultural and institutional differences may influence leadership practices and ethical climates across healthcare settings. Future research should, therefore, replicate the study across multiple hospitals, regions, and cultural contexts using more representative sampling approaches where feasible.

Third, the use of self‐report measures raises the possibility of common method variance and social desirability bias. Although procedural and statistical remedies were applied, participants may still have overstated moral courage or underreported negative leadership experiences. Future studies could strengthen validity by incorporating multisource data, peer or supervisor evaluations, observational methods, or behavioral indicators of ethical conduct.

In addition, the model focused primarily on leadership, EDI, ethical climate, and compassion fatigue, while other potentially important factors were not examined. Individual variables such as resilience, moral identity, and professional calling, as well as organizational conditions including workload, staffing levels, and institutional support, may also influence nurses’ moral courage. Future research could incorporate these variables to provide a more comprehensive understanding of ethical behavior in nursing.

Finally, although PLS‐SEM was appropriate for the exploratory and complex nature of the proposed model, future studies may benefit from combining PLS‐SEM with covariance‐based SEM to further validate the robustness of the findings and improve measurement precision.

## 6. Conclusion

This study examined how ethical, empowering, and AUL influence nurses’ moral courage through EDI, with PEC and compassion fatigue acting as moderating factors. The findings showed that ethical and empowering leadership positively promoted moral courage, whereas AUL had a negative effect.

EDI emerged as a key mechanism linking leadership to moral action, while ethical climate and compassion fatigue shaped the strength of these relationships. Specifically, supportive ethical climates strengthened the positive effects of leadership, whereas compassion fatigue weakened them.

The study contributes to ethical decision‐making research by demonstrating how leadership, ethical cognition, organizational context, and psychological well‐being jointly shape ethical behavior in nursing. Practically, healthcare organizations should foster ethical and empowering leadership, strengthen ethical climates, and reduce compassion fatigue to better support nurses in delivering ethically grounded and patient‐centered care.

## Author Contributions

Mei Xie: conceptualization, funding acquisition, project administration, supervision, and writing–review and editing; Zefeng Shao: formal analysis, data curation, and visualization; Huimin Su and Quanyi Long: writing–review and editing; Nik Mohd Hazrul Nik Hashim: validation and methodology; Kifayat Nahiyan Rafi: validation, methodology, and visualization; Yuanyuan Zou: conceptualization, investigation, methodology, writing–original draft, and funding acquisition. Mei Xie and Zefeng Shao are co‐first authors.

## Funding

This work was supported by the China Scholarship Council (No. 202208530084 from Yuanyuan Zou and No. 202207000022 from Mei Xie). Open access publishing facilitated by Universita degli Studi di Roma La Sapienza, as part of the Wiley ‐ CRUI‐CARE agreement.

## Ethics Statement

Ethical approval was obtained from the Ethics Committee of Kunming Medical University Affiliated Hospital (Approval No. KMMU2025MEC175). All participants provided informed consent before data collection, and all procedures complied with the principles of the Declaration of Helsinki.

## Conflicts of Interest

The authors declare no conflicts of interest.

## Data Availability

The data that support the findings of this study are available from the corresponding author upon reasonable request.

## Appendix

**TABLE A1:** Construct items.

Items	Reference
Ethical leadership	[46]
1. My leader places great importance on ethics and moral values.	
2. My leader clearly communicates standards of ethical behavior to team members.	
3. My leader leads by example in decisions and behaviors, demonstrating a moral role model.	
4. My leader is consistent in words and actions, practicing the values they proclaim.	
5. My leader is fair and impartial in assigning tasks.	
6. My leader is trustworthy and fulfills promises and responsibilities.	
7. My leader is able to admit mistakes and is willing to take responsibility.	
8. My leader regards honesty and integrity as important personal values.	
9. My leader sets an example through dedication to the organization and a spirit of self‐sacrifice.	
10. My leader opposes improving performance through unethical means.	
11. My leader is fair and objective in performance evaluations and reward allocations.	
Empowering leadership	[24]
1. My leader encourages team members to express ideas and suggestions.	
2. My leader attentively listens to our team’s opinions and suggestions.	
3. My leader adopts our team’s suggestions to make decisions that affect us.	
4. My leader encourages our team members to solve problems together.	
5. My leader encourages team members to exchange information with each other.	
6. My leader provides necessary assistance to team members.	
7. My leader supports our team’s work efforts.	
Authoritarian leadership	[29]
1. My leader requires me to fully obey his/her instructions.	
2. Regardless of the size of the matter, my leader personally makes all decisions.	
3. In every meeting, the final decision‐making power is always held by my leader.	
4. My leader always presents themselves in a commanding manner in front of employees.	
5. When working with my leader, I often feel pressured.	
6. My leader implements strict disciplinary management over subordinates.	
7. When we fail to complete tasks, my leader reprimands us.	
8. My leader emphasizes that our nursing team’s performance must be superior to other departments in the entire hospital.	
9. We must complete work according to the rules he/she has established, otherwise we will receive severe punishment.	
Moral courage behavior	[53]
1. Even in difficult circumstances, I support suffering patients through genuine presence and companionship.	
2. Even under limited conditions, I strive to establish authentic interpersonal connections with patients.	
3. Even when it is challenging, I discuss illness‐related fears with patients.	
4. Even when faced with difficulties, I persist in acting according to professional ethical principles.	
5. Even with insufficient resources, I strive to ensure patients receive good care.	
6. If a patient’s care rights are threatened, I proactively initiate discussions.	
7. If I observe others exhibiting unprofessional or dishonest behavior at work, I take action.	
8. If someone attempts to cover up obvious care errors, I adhere to professional ethical principles.	
9. If I witness unethical behavior in others, I respond or report it.	
10. If I observe obvious deficiencies in others’ care practices, I point them out.	
Ethical dilemma identification	[52]
1. I am able to carry out my work in accordance with my own moral standards.	
2. I am able to engage in nursing work in a manner that I consider morally correct.	
3. I receive support in my work to perform ethical behaviors.	
4. I receive assistance in my work that enables me to practice in a morally correct way that I believe is right.	
Perceived ethical climate	[49]
1. The hospital places greater emphasis on the efficiency of nursing work rather than the process.	
2. The hospital encourages nurses to prioritize organizational benefits.	
3. The hospital encourages nurses to act in accordance with the law under any circumstances.	
4. The hospital expects nurses to prioritize the overall interests of the organization.	
5. The hospital emphasizes that nurses must obey its management rules and regulations.	
6. The hospital values the spirit of collaboration in nursing teams.	
7. The hospital management tends to evaluate nursing behaviors from the perspective of economic benefits.	
8. The hospital values nurses′ investments in improving employee welfare.	
9. The hospital emphasizes that all nursing decisions should be centered on the organization’s goals.	
10. The hospital advocates that every patient should receive personalized care.	
11. The hospital encourages nurses to find the most effective solutions in the shortest time.	
Compassion fatigue	[51]
1. I have difficulty separating work from personal life.	
2. When work pressure is high, I try to regulate my emotions with humor.	
3. I make time for myself to relax through methods such as meditation, massage, or personal rest.	
4. After experiencing a patient’s death in the ward, I cherish the time for communication and companionship with leaders.	
5. I often bring negative emotions from work home, affecting family life.	
6. I proactively make time to temporarily leave work.	
7. When facing situations of loss, grief, or death, I have my own fixed coping methods or rituals.	
8. I have established supportive professional interpersonal relationships.	
9. I am aware of the need to maintain appropriate personal boundaries between myself and patients.	
10. Even when patients experience death or trauma, I can still see the value in this work.	
